# Prognostic factors for changes in the timed 4-stair climb in patients with Duchenne muscular dystrophy, and implications for measuring drug efficacy: A multi-institutional collaboration

**DOI:** 10.1371/journal.pone.0232870

**Published:** 2020-06-18

**Authors:** Nathalie Goemans, Brenda Wong, Marleen Van den Hauwe, James Signorovitch, Gautam Sajeev, David Cox, John Landry, Madeline Jenkins, Ibrahima Dieye, Zhiwen Yao, Intekhab Hossain, Susan J. Ward, the Collaborative Trajectory Analysis Project (cTAP)

**Affiliations:** 1 Department of Child Neurology, University Hospitals Leuven, Leuven, Belgium; 2 Department of Pediatrics, University of Massachusetts Medical School, Worcester, MA, United States of America; 3 Analysis Group Inc., Boston, Massachusetts, United States of America; 4 The Collaborative Trajectory Analysis Project, Cambridge, Massachusetts, United States of America; 5 Eli Lilly and Company, Indianapolis, Indiana, United States of America; 6 Eli Lilly and Company, Toronto, Ontario, Canada; 7 Analysis Group Inc., London, United Kingdom; Cleveland Clinic, UNITED STATES

## Abstract

The timed 4-stair climb (4SC) assessment has been used to measure function in Duchenne muscular dystrophy (DMD) practice and research. We sought to identify prognostic factors for changes in 4SC, assess their consistency across data sources, and the extent to which prognostic scores could be useful in DMD clinical trial design and analysis. Data from patients with DMD in the placebo arm of a phase 3 trial (Tadalafil DMD trial) and two real-world sources (Universitaire Ziekenhuizen, Leuven, Belgium [Leuven] and Cincinnati Children's Hospital Medical Center [CCHMC]) were analyzed. One-year changes in 4SC completion time and velocity (stairs/second) were analyzed. Prognostic models included age, height, weight, steroid use, and multiple timed function tests and were developed using multivariable regression, separately in each data source. Simulations were used to quantify impacts on trial sample size requirements. Data on 1-year changes in 4SC were available from the Tadalafil DMD trial (n = 92) Leuven (n = 67), and CCHMC (n = 212). Models incorporating multiple timed function tests, height, and weight significantly improved prognostic accuracy for 1-year change in 4SC (R^2^: 29%-36% for 4SC velocity, and 29%-34% for 4SC time) compared to models including only age, baseline 4SC and steroid duration (R^2^:8%-17% for 4SC velocity and 2%-13% for 4SC time). Measures of walking and rising ability contributed important prognostic information for changes in 4SC. In a randomized trial with equal allocation to treatment and placebo, adjustment for such a prognostic score would enable detection (at 80% power) of a treatment effect of 0.25 stairs/second with 100–120 patients, compared to 170–190 patients without prognostic score adjustment. Combining measures of ambulatory function doubled prognostic accuracy for 1-year changes in 4SC completion time and velocity. Randomized clinical trials incorporating a validated prognostic score could reduce sample size requirements by approximately 40%. Knowledge of important prognostic factors can also inform adjusted comparisons to external controls.

## Introduction

Duchenne muscular dystrophy (DMD) is a progressive, debilitating neuromuscular disorder occurring in approximately 15.9 to 19.5 per 100,000 live births, based on estimates from the United States and United Kingdom [1, 2]. DMD results in progressive muscle-wasting, loss of ambulation during adolescence, and death by early adulthood [3]. Over the last decade substantial progress has been made to identify and develop targeted therapies to treat the underlying cause of DMD [4]. However, although several drugs [5–9] have been tested in late-phase placebo-controlled trials, conditional or full regulatory approval has thus far been secured for only two novel drugs in the ambulatory setting: ataluren for nonsense mutation DMD [10], and eteplirsen for exon 51 skippable mutations [11].

A major challenge in the design of DMD clinical trials, and in the interpretation of their results, is heterogeneity in patients' rates of disease progression [5, 6, 8, 9]. To address this, investigators have sought to enrich trial populations for patients with less variable prognoses over the duration of a trial. Such enrichment efforts have been implemented through the use of selected patient characteristics, in particular age, duration of steroid use and baseline performance on the primary functional endpoint, to define inclusion/exclusion criteria and to stratify randomization in clinical trials. However, even in trials incorporating these approaches, observed variability in functional outcomes remains large, spanning improvement in function for some patients to complete loss of function in others.

At the same time, evidence has emerged that additional patient characteristics, particularly those measuring different aspects of ambulatory function, have additive value as important prognostic factors. Performance on the timed rise from supine has been associated with changes on the six minute walk distance (6MWD) and disease progression in general [12, 13]. In addition, our earlier work has demonstrated that a composite score based on multiple baseline measures of function more than doubled prognostic accuracy for changes in 6MWD compared to predictions based on age, baseline 6MWD, and steroid use [12, 14].

Use of prognostic factors to inform clinical trial design is well-established in many therapeutic areas, including, for example, cardiovascular disease [15], renal disease [16], and oncology [17] trials. The use of prognostic enrichment has also been noted as a potentially useful strategy for clinical trials in guidance issued by the Food and Drug Administration (e.g., see sections IV and V of [18]). The stronger the prognostic factors, the higher their value for trial design in terms of increasing power and decreasing sample size requirements. Better understanding of prognostic factors could be especially valuable for trials in DMD due to challenges associated with heterogeneity in patients' progression over time and the limited patient populations available to participate in trials.

Since DMD is characterized by progressive deficits in muscle strength, the timed 4-stair climb (4SC) has been used as an important assessment in clinical practice and in clinical trials [5–7, 9, 19]. In the present study we assess prognostic factors for 1-year changes in 4SC performance, develop a preliminary composite prognostic score, and assess consistency of prognostic performance across data sources. We also quantify the impacts that use of the prognostic score would have on sample size requirements in clinical trials. This study was conducted within the collaborative Trajectory Analysis Project (cTAP), a pre-competitive collaboration of drug developers, clinical experts and registries, patient advocacy groups, and data scientists engaged in research to improve drug evaluations in DMD.

## Methods

### Data sources and study population

This study used data shared with cTAP from three different sources: placebo arm data from Eli Lilly's phase 3 trial of tadalafil in patients with DMD (NCT01865084), and real-world data from DMD centers in Leuven, Belgium (Leuven) and at the Cincinnati Children's Hospital Medical Center (CCHMC) in Cincinnati, Ohio. At the time of this study, these constituted all data sources accessed by cTAP with available data on the 4SC test.

#### Tadalafil DMD trial

The Tadalafil DMD Study Group Trial (Tadalafil DMD Trial) was a randomized, double-blind, placebo-controlled phase 3 trial of tadalafil, which enrolled ambulatory boys with DMD aged 7 to 14 years, who had at least 6 months of steroid use prior to trial recruitment, and baseline 6MWD between 200 and 400 meters [5]. Patients were randomly assigned to receive placebo, low dose tadalafil (0.3 mg/kg), or high dose tadalafil (0.6 mg/kg) daily for 48 weeks [5]. Trial assessments, including timed function tests, occurred every 12 weeks. Only those patients randomized to placebo were included in the present study. The study protocol and consent forms for the use of data from Eli Lilly's phase 3 trial of tadalafil in patients with DMD were approved by the institutional review/ethics boards at each participating medical center and conducted in accordance with the Declaration of Helsinki and other international ethics guidelines. The phase 3 trial data used in this study were de-identified and thus no institutional review board (IRB) approval was required for the de-identified data used in this analysis.

#### Leuven

Data were collected from boys diagnosed with DMD who were routinely monitored in the pediatric neurology clinic at Universitaire Ziekenhuizen in Leuven, Belgium. This ongoing study has been approved by Ethische Commissie Onderzoek, the Ethics Committee of the University Hospitals Leuven, and was conducted in accordance with the Declaration of Helsinki. Written consent from the guardians of each participant was obtained. The database available for the present study included 158 boys with DMD and clinic visits as recent as February 2017. Assessments of timed function tests occurred approximately every 6 months, and included the timed 4SC, 10-meter walk/run, and rise from supine, as well as concurrent assessments of height, weight, and steroid use.

#### CCHMC

Natural history data were obtained from electronic medical records of patients with DMD receiving care at CCHMC. The data was fully de-identified and was collected under an IRB-approved clinic registry, CCHMC IRB #1, which captures clinical data from clinic visits with informed consent from patients and care givers to participate in the clinic registry at CCHMC. The database available for the present study spanned the years 2003 to 2016 and included 480 boys diagnosed with DMD. Functional assessments occurred every 6 months to 1-year, and included timed 4SC, timed 30-foot walk/run, and timed sit to stand.

### Study measures

#### Outcomes

The primary outcome measures in this study were the annualized changes in 4SC velocity (Δ4SC velocity) and 4SC completion time (Δ4SC time) over an approximately 1-year period (8 to 16 months). While 4SC completion time (recorded in seconds taken to complete the test) may be more clinically interpretable, 4SC velocity (measured in stairs/second) may have desirable statistical properties. It was not the purpose of this study to assess the relative statistical value of these measurement scales. Both have been considered in planned analyses of DMD clinical trials. Both measures were investigated here to assess sensitivity of prognostic factors to choice of 4SC measurement scale.

Annualized changes in 4SC were calculated by dividing the change observed between two assessments by the elapsed time (in years) between those assessments. Patients who could not complete the 4SC due to loss of ability to climb stairs had their velocities set to zero and their completion times set to 12 seconds; completion times exceeding 12 seconds were also truncated to 12 seconds, as this reflects the typical maximum recording of 4SC completion times in real-world clinical practice. Sensitivity analyses using a threshold of 30 seconds, which has been used as the maximum recording time in some clinical trials [6, 7] were also conducted. The analyses on the velocity scale also serve as a sensitive analysis for the issue of truncation of completion times, since, on the velocity scale, larger completion times are translated into smaller velocities approaching zero. The current study is not intended to recommend appropriate cut-off times for clinical trials or clinical practice, but rather to assess the sensitivity of prognostic factors to different cutoff times. 4SC assessments that were not performed for other reasons (e.g., bone fracture) were considered missing.

There were some differences across data sources in how 4SC times were assessed and recorded, and in availability of data on loss of ability to climb stairs. In Leuven and the Tadalafil DMD Trial placebo arm, task completion occurred when the patient's second foot reached the last step, whereas in CCHMC task completion occurred when the patient's leading foot reached the last step. Additionally, the inability to complete the 4SC due to the loss of ability to climb stairs was not captured in clinical practice data available from CCHMC. Consequently, the 4SC time and velocity data from CCHMC reflects data only from patients who were able to complete the test. In contrast, the inability to complete the 4SC test was explicitly recorded at all visits in Leuven and the Tadalafil DMD Trial placebo arm.

#### Prognostic factors

In all three data sources prognostic factors assessed included demographics and vitals (age, height, weight, body mass index [BMI]), duration of steroid use (≥1 year vs. <1 year), current deflazacort use (yes/no), and timed function tests. Apart from 4SC, the available timed function tests differed across data sources. The 10 meter walk/run and timed rise from supine were available in Leuven and the Tadalafil DMD Trial; in CCHMC, the 30-foot walk/run and timed sit to stand were available in lieu of these two tests. Patients (in the Tadalafil DMD Trial and Leuven) who were unable to complete the 10-meter walk/run and rise from supine tests, or those who took longer than 12 seconds to complete, had their times set to 12 seconds for analysis. Velocities for these timed function tests were set to zero for patients who had lost the function being assessed by the test.

### Study design

**Fig 1:**
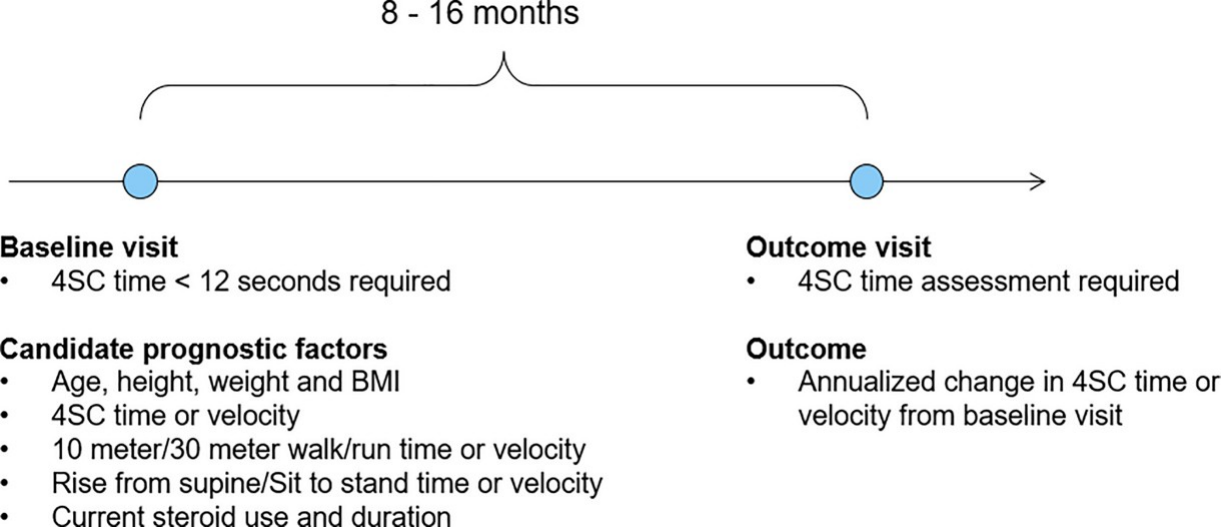
Study design. Changes in timed 4-stair climb (4SC) were evaluated over approximate 1-year follow-up intervals (8–16 months). 4SC, 4-stair climb; BMI, body mass index.

Changes in 4SC were studied over intervals of follow-up that were approximately 1-year in length **(Fig 1).** For each data source, we identified all intervals (i.e. pairs of clinic visits) meeting each of the following criteria: 1) the first clinic visit in the pair, defined as the *baseline visit*, had 4SC time < 12 seconds and information available for all prognostic factors of interest; 2) the baseline visit and a follow-up visit, defined the *outcome visit*, were separated by approximately 1 year (8 to 16 months); 3) 4SC was assessed at both the baseline and outcome visits. Separate study samples were constructed in this fashion for each data source. Patients from the Tadalafil DMD Trial contributed one ~1-year interval each, corresponding to the approximately 1-year between the baseline and 48-week assessments in the trial. Patients from Leuven and CCHMC could have multiple pairs of ~1-year intervals meeting the aforementioned criteria. For these patients, all non-overlapping ~1-year intervals were included in the analyses. The outcome visit for one interval was allowed to serve as the baseline visit for a subsequent interval, but further overlap was disallowed.

Δ4SC time = (4SC time at outcome visit - 4SC time at baseline visit)/ time in years between outcome and baseline visits. Δ4SC time > 0 indicates worsened performance; Δ4SC time < 0 improved performance.

Δ4SC velocity = (4SC velocity at outcome visit - 4SC velocity at baseline visit)/ time in years between outcome and baseline visits. Δ4SC velocity > 0 indicates improved performance; Δ4SC velocity < 0 indicates worsened performance.

### Statistical analyses

Baseline characteristics were summarized for each data source using means and standard deviations (SD) for continuous variables and counts and percentages for categorical variables. Observed Δ4SC velocity and Δ4SC completion times were also summarized for each data source.

#### Fitting prognostic models

Multivariable regression models for the Δ4SC velocity were fit in each data source to assess the importance of the candidate prognostic factors. Three models were fit: (1) a *base model* that contained only age, baseline 4SC velocity, and duration of steroid use; (2) an *intermediate model* that incorporated current deflazacort use, measures of walk/run and rising function, in addition to the factors in the base model, and (3) a *full model* that incorporated height, weight, and BMI, in addition to the factors included in the intermediate model. In all models, generalized estimating equations, with an exchangeable covariance structure were used to account for repeated measures in Leuven and CCHMC. A similar set of models were fit for 4SC completion time in each data source, with the corresponding timed function test completion times replacing velocities used as baseline predictors in the intermediate and full models.

#### Assessing prognostic value

The prognostic value of each model was measured by calculating the root mean squared prediction error, which was computed as the SD of the difference between observed and model-predicted Δ4SC velocity. To visually assess the performance of each model, scatterplots of the observed versus model-predicted Δ4SC velocity were generated. R^2^ values were also calculated to measure the percentage of variation in Δ4SC velocity explained by each model. Box plots of the observed Δ4SC velocity, stratified by quartiles of predicted change, were used to assess model calibration. Finally, contributions of predictors to the overall prognostic value of the model was assessed by estimating R^2^ values for multivariable models obtained by separately adding each predictor to the base model, and separately removing each predictor from the full model. Because the rise from supine and 10-meter walk/run were not assessed in data from CCHMC, these predictors were replaced with sit to stand and 30-foot walk/run tests, respectively. Similar analyses were carried out for Δ4SC time.

#### Sensitivity analyses

Two sets of sensitivity analyses were conducted. First, the analyses were repeated with 4SC completion times for patients who had lost the ability to climb stairs set to 30 seconds. In this sensitivity analysis, patients with 4SC completion times exceeding 30 seconds had these times truncated to 30 seconds (rather than 12 seconds as in the primary analyses). Second, the models were re-fit using 1-year intervals defined based on visits 10 to 14 months apart rather than 8 to 16 months apart as in the primary analyses.

#### Impacts on trial sample size

Simulations were used to quantify the extent to which use of a prognostic score could reduce sample size requirements for randomized controlled trials in DMD. These simulations assumed that changes in 4SC velocity are normally distributed, hypothesized a treatment effect on mean Δ4SC velocity of 0.25 stairs/second, assumed a type I error rate of 0.05, and considered different scenarios for the level of variability of Δ4SC velocity (SD of Δ4SC velocity either 0.5, 0.6, or 0.7 stairs/second). For each scenario, power to detect the treatment effect was estimated for two possible statistical analyses: 1) an unadjusted analysis and 2) an analysis adjusting for a baseline prognostic score with an R^2^ of 0.35.

## Results

### Baseline sample characteristics

Δ4SC velocity was available from 92 patients (contributing 92 ~1-year intervals) from the Tadalafil DMD Trial, 67 patients (235 ~1-year intervals) from Leuven, and 212 patients (543 ~1-year intervals) from CCHMC **(Table 1)**. Baseline characteristics are summarized in **Table 2**. Patient demographics were generally similar across all three sources at the start of the ~1-year intervals analyzed. Duration of steroid use was longer in CCHMC and in the Tadalafil DMD Trial than in Leuven. Patients in CCHMC and Leuven predominantly received deflazacort at baseline; in the Tadalafil DMD Trial, steroid use at baseline was evenly split between deflazacort and prednisone. Patients in the Tadalafil DMD Trial had slightly worse function at baseline, on average, as indicated by their lower North Star Ambulatory Assessment (NSAA) total score and longer completion times (lower velocities) on the available timed function tests.

**Table 1:** Sample selection in each data source.

	Tadalafil DMD Trial	Leuven	CCHMC
**Step 1: All patients**	116	158	480
**Step 2: Patients with at least 2 4SC visits**	116	90	261
**Step 3: Patients with 4SC visits approximately 1-year apart (and total # of such intervals)**	111 (111)	70 (1011)	230 (877)
**Step 4: Patients and intervals meeting eligibility criteria and having non-missing data on prognostic factors at baseline visit of the interval**	92 (92)	67 (711)	213 (684)
**Step 5: Patients in Step 4 with non-overlapping intervals**	92 (92)	67 (235)	212 (543)

4SC, 4-stair climb; CCHMC, Cincinnati Children's Hospital Medical Center; DMD, Duchenne muscular dystrophy.

Steps 3, 4, and 5 show number of patients (intervals) meeting each criterion. In step 3, for the Leuven and CCHMC databases, ~1-year intervals were identified on the basis of having 4SC visits 8–16 months apart. For the Tadalafil DMD trial, the ~1-year intervals reflect the ~1-year between the baseline and 48-week assessments in the trial.

**Table 2:** Baseline sample characteristics in each data source.

	Tadalafil DMD Trial Placebo Arm	Leuven	CCHMC
**Number of ~1-year intervals**	92	235	543
**Number of patients**	92	67	212
**Demographics, mean ± SD, median [range]**			
Age (years)	9.36 ± 1.79 9.13 [7.00, 14.58]	9.10 ± 2.73 8.84 [3.17, 17.42]	8.81 ± 2.73 8.51 [2.42, 18.54]
Height (cm)	125.17 ± 8.73 126.00 [108.80, 147.20]	122.08 ± 10.60 122.50 [92.60, 154.00]	120.81 ± 12.24 120.20 [80.00, 167.30]
Weight (kg)	30.92 ± 8.86 28.40 [17.20, 61.40]	28.36 ± 9.86 25.40 [14.20, 70.00]	28.85 ± 10.54 26.75 [12.20, 88.94]
BMI (kg/m^2^)	19.54 ± 4.31 18.01 [13.16, 35.02]	18.53 ± 3.77 17.43 [12.10, 32.22]	19.20 ± 3.72 18.44 [12.73, 37.21]
**Steroid use**			
Steroid duration (months), mean ± SD	39.19 ± 24.42 35.83 [6.83, 112.46]	29.11 ± 26.71 23.52 [0.00, 121.43]	43.05 ± 31.36 40.32 [0.00, 196.32]
Current deflazacort, n (%)	44 (47.83)	178 (75.74)	448 (82.50)
** Current steroid use, n (%)**	92 (100%)	198 (84.26)	499 (91.90%)
**Ambulatory and timed function tests, mean ± SD**			
NSAA total score	22.13 ± 6.03 22.50 [9, 34]	24.54 ± 6.43 26.00 [10, 33]	24.72 ± 5.93 26.00 [7, 34]
6MWD (meters)	348.71 ± 39.29 355.00 [216, 410]	365.96 ± 87.76 376.00 [0, 620]	-
4-stair climb, time (seconds)	5.1 ± 2.3 4.6 [1.5, 11.6]	3.8 ± 2.4 3.2 [1.1, 11.9]	2.6 ± 1.5 2.2 [0.8, 10.4]
4-stair climb, velocity (stairs/second)	0.97 ± 0.46 0.87 [0.34, 2.67]	1.42 ± 0.75 1.27 [0.34, 3.77]	1.96 ± 0.91 1.82 [0.38, 5.00]
Rise from supine, time (seconds)	7.6 ± 3.8 6.6 [1.8, 17.2]	6.5 ± 8.4 4.5 [1.4, 108.0]	-
Rise from supine, velocity (1/seconds)	0.15 ± 0.11 0.13 [0.00, 0.56]	0.22 ± 0.14 0.21 [0.00, 0.74]	
10-meter walk/run, time (seconds)	6.4 ± 1.7 6.2 [2.9, 10.9]	5.5 ± 2.3 4.9 [1.5, 20.4]	-
10-meter walk/run, velocity (meters/second)	1.69 ± 0.48 1.61 [0.92, 3.45]	2.07 ± 0.69 2.04 [0.49, 6.54]	
Sit to stand, time (seconds)	-	-	2.9 ± 2.3 2.1 [0.9, 21.6]
Sit to stand, velocity (1/seconds)	-	-	0.46 ± 0.20 0.48 [0.05, 1.11]
30-foot walk/run, time (seconds)	-	-	4.4 ± 1.1 4.1 [2.2, 10.7]
30-foot walk/run, velocity (feet/second)	-	-	7.28 ± 1.64 7.32 [2.80, 13.64]

6MWD, 6-minute walk distance; BMI, body mass index; CCHMC, Cincinnati Children's Hospital Medical Center; cm, centimeters; DMD, Duchenne muscular dystrophy; kg, kilograms; m^2^, meters squared; n, number of intervals; NSAA, North Star Ambulatory Assessment; SD, standard deviation.

Timed function tests in baseline table are calculated without imputation.

### Observed Δ4SC velocity and Δ4SC time

4SC velocity declined over the ~1-year period in all three data sources: mean Δ4SC velocity was -0.06 stairs/second (SD = 0.65) in Leuven, -0.18 stairs/second (SD = 0.41) in the Tadalafil DMD Trial, and -0.12 stairs/second (SD = 0.56) in CCHMC. Correspondingly, completion times for 4SC increased over the ~1-year period in all three data sources: mean Δ4SC time in Leuven was 0.7 seconds (SD: 2.2, range: [-4.3 to 10.2]) 2.0 seconds (SD: 2.9, range: [-7.4, 8.6]) in the Tadalafil DMD Trial, and 0.5 seconds (SD: 1.5, range: [-4.5, 9.2]) in CCHMC. The ability to complete 4SC was lost in 17 (7.2%) of the 235 intervals from Leuven, and 6 (6.5%) of the 92 intervals from the Tadalafil DMD Trial. As noted above, this information was not available in data from CCHMC.

### Models for Δ4SC velocity time and Δ4SC time

#### Δ4SC velocity

Results from the base and full multivariable models for Δ4SC velocity are presented in **Table 3.** Model coefficients and R-squared values from the intermediate model for Δ4SC velocity were similar to the full model and are shown in **S1 Table.** Overall, the base model explained 17% and 16% of the variation of **Δ**4SC velocity in Leuven and CCHMC patients, respectively. In these data sources, older age and longer duration of steroid use and higher baseline 4SC velocity were associated with declines in 4SC velocity over the ~1-year period. Among patients from the Tadalafil DMD Trial, 8% of the variation in **Δ**4SC velocity was explained by the predictors included in the base model, with none of them being statistically significant.

**Table 3:** Models for Δ4SC velocity in each data source.

	Tadalafil DMD Trial Placebo Arm (n = 92)		Leuven (n = 235)		CCHMC (n = 543)	
	Base model	Full model	Base model	Full model	Base model	Full model
	Coefficient (95% CI)	Coefficient (95% CI)	Coefficient (95% CI)	Coefficient (95% CI)	Coefficient (95% CI)	Coefficient (95% CI)
**Intercept**	-1 (-1.88, -0.13)*	5.53 (-0.07, 11.13)	0.98 (0.66, 1.29)	0.74 (-3.19, 4.66)	0.77 (0.55, 0.99)***	3.02 (1.15, 4.89)**
**Age (years)**	0.03 (-0.02, 0.08)	0.04 (-0.02, 0.09)	-0.08 (-0.11, -0.05)***	-0.03 (-0.07, 0.01)	-0.06 (-0.08, -0.04)***	-0.05 (-0.09, -0.02)**
**Steroids ≥ 1 year 1 vs. 0**	0.26 (-0.05, 0.56)	0.42 (0.16, 0.69)**	-0.15 (-0.33, 0.03)	-0.29 (-0.47, -0.11)**	-0.16 (-0.28, -0.04)**	-0.24 (-0.37, -0.10)***
**Timed 4-stair climb velocity**	0.29 (-0.09, 0.66)	-0.17 (-0.66, 0.31)	-0.16 (-0.25, -0.07)***	-0.6 (-0.74, -0.46)***	-0.12 (-0.16, -0.07)***	-0.42 (-0.50, -0.34)***
**Current deflazacort 1 vs. 0**		0.16 (-0.01, 0.33)		0.16 (-0.02, 0.34)		0.01 (-0.12, 0.14)
**Timed 10-meter walk/run velocity**		0.22 (-0.07, 0.50)		0.27 (0.17, 0.37)***		-
**Timed rise from supine velocity**		1.41 (0.25, 2.56)*		1.83 (1.13, 2.53)***		-
**Timed 30-foot walk/run velocity**	-	-	-	-		0.13 (0.08, 0.18)***
**Timed sit to stand velocity**	-	-	-	-		0.72 (0.31, 1.13)***
**BMI (kg/m^2^)**		-0.19 (-0.33, -0.05)**		0.02 (-0.08, 0.12)		-0.1 (-0.14, -0.05)***
**Height (cm)**		-0.05 (-0.10, -0.01)*		-0.01 (-0.04, 0.03)		-0.02 (-0.04, -0.01)**
**Weight (kg)**		0.11 (0.03, 0.20)**		-0.01 (-0.06, 0.05)		0.06 (0.03, 0.08)***
**Model R^2^ (adjusted R^2^)**	0.08 (0.05)	0.29 (0.21)	0.17 (0.16)	0.36 (0.33)	0.16 (0.16)	0.30 (0.29)
**RMSE**	0.39	0.35	0.59	0.52	0.51	0.47

Δ4SC, annualized change in 4-stair climb; BMI, body mass index; CCHMC, Cincinnati Children's Hospital Medical Center; CI, confidence interval; cm, centimeters; kg, kilograms; m^2^, meters squared; n, number of participants; RMSE, root-mean squared error. Statistical significance: *** p < 0.001, ** p < 0.01, * p <0.05

In all three data sources, the full model, additionally including timed function tests (rise from supine, 10 meter walk/run, or sit to stand and 30 foot walk/run) and additional patient characteristics (current steroid use, height, weight, and BMI), explained a greater proportion of the variation in Δ4SC velocity (29% in the Tadalafil DMD Trial, 36% in Leuven, and 30% in CCHMC). In the full models fit in Leuven and CCHMC, longer duration of steroid use and greater velocities of 10-meter walk/run and rise from supine (or 30-foot walk/run and sit to stand) at baseline were associated with greater improvements in 4SC velocity. Higher 4SC velocity at baseline was associated with a decrease in **Δ**4SC velocity over the 1-year period.

Scatterplots of the observed versus model-predicted **Δ**4SC velocity, and box plots of the observed Δ4SC velocity, stratified by quartiles of predicted change illustrate the improvement in prediction of **Δ**4SC velocity with the addition of the prognostic factors in the full model (**S1 and S2 Figs**). Models were further evaluated by inspection of residuals plotted relative to predicted values, which were consistent with adequate model specification. In addition, use of generalized estimating equations rendered the models robust to potential non-normality of residuals. The contributions of each prognostic factor when removed from the full model, added separately to the base model, and studied in isolation are summarized in **S2A–S2C Table**. In general, the addition of velocities for 10-meter walk/run, rise from supine (or, 30 foot walk/run, sit to stand in CCHMC), and current deflazacort use to the base model resulted in the greatest increases in R^2^ while removing baseline 4SC velocity from the full model resulted in the largest decreases in R^2^.

**Fig 2:**
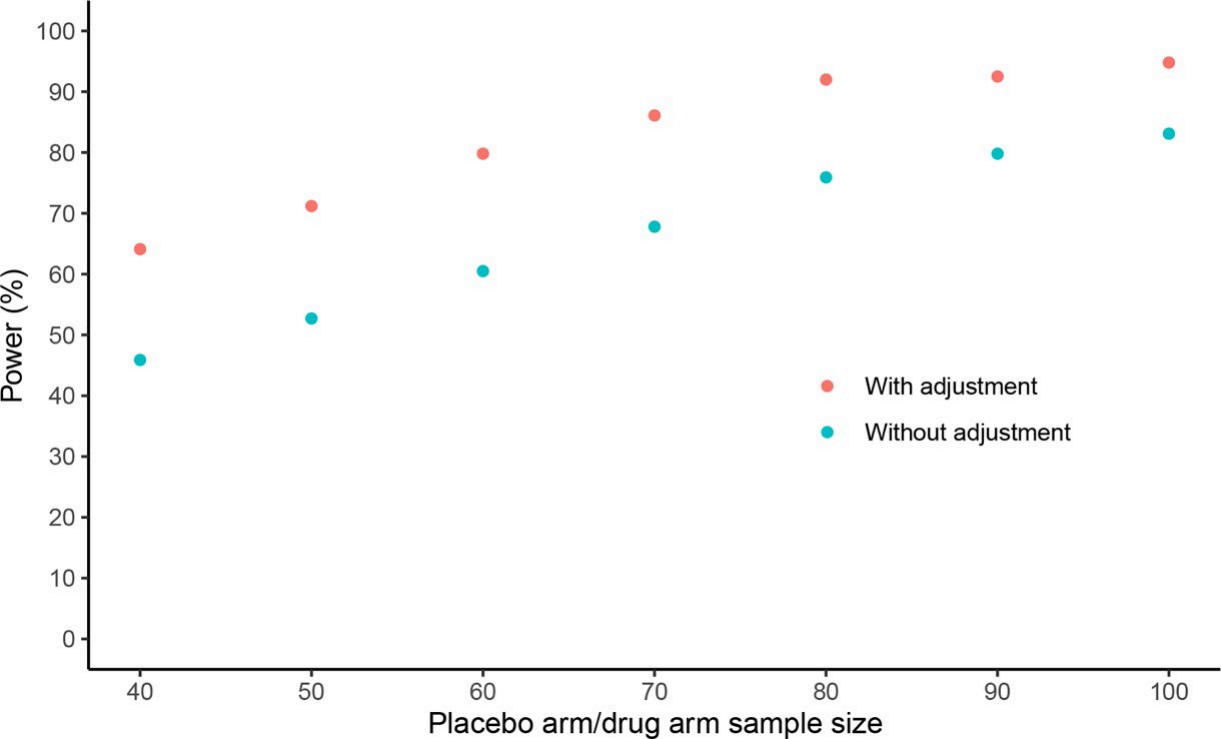
Power to detect a treatment effect of 0.25 stairs/second with and without adjustment for prognostic score under different trial arm sample sizes, assuming the SD of Δ4SC velocity is 0.6. Δ4SC, annualized change in 4-stair climb; SD, standard deviation. Δ4SC velocity = (4SC velocity at outcome visit - 4SC velocity at baseline visit)/ time in years between outcome and baseline visits. Δ4SC velocity > 0 indicates improved performance; Δ4SC velocity < 0 indicates worsened performance. R-squared due to prognostic model assumed to be 0.35 in all scenarios. While a SD of Δ4SC velocity of 0.6 was used for this illustration, a similar pattern of greater power to detect a treatment effect when adjustment is made for the prognostic score also holds for lower and higher SDs of Δ4SC velocity (S3 and S4 Figs).

In a randomized trial with equal allocation to treatment and placebo arms, baseline adjustment for such a prognostic score would enable a treatment effect of 0.25 stairs/second on Δ4SC velocity to be detected with approximately 120 total patients, compared to 180 total patients without use of the prognostic score (at 80% power) **(Fig 2).** Power gains with adjustment for prognostic score under additional scenarios are shown in **S3 and S4 Figs**.

#### Δ4SC time

Results from the base and full multivariable models for Δ4SC time are presented in **S3 Table**. Overall, the base model explained 13% and 11% of the variation of **Δ**4SC time in patients from Leuven and CCHMC, respectively, with older age and longer duration of steroid use associated with worsening outcomes. In the Tadalafil DMD Trial, the base model explained 2% of the variation of **Δ**4SC time with none of the predictors being statistically significant. Results of the intermediate model for Δ4SC time are shown in **S4 Table.**

Compared with the base model, the full model explained a greater proportion of the variation of **Δ**4SC time from all three data sources (29% in the Tadalafil DMD Trial, 34% in Leuven, and 34% in CCHMC). Associations of the predictors with **Δ**4SC time were generally very similar to those seen for **Δ**4SC velocity. The contributions of each prognostic factor when removed from the full model, added separately to the base model, and studied in isolation are summarized in **S5A–S5C Table**. In general, the addition of 10-meter walk/run, rise from supine, and current deflazacort use to the base model resulted in the greatest increases in R^2^ while removing baseline 4SC completion time from the full model resulted in the largest decreases in R^2^.

#### Sensitivity analyses

Results from both sets of sensitivity analyses were very similar to the primary analyses in all three data sources: R^2^ for models for each set of analyses are summarized in **S6A and S6B Table**.

## Discussion

This study identified prognostic factors for ~1-year change in 4SC completion time and velocity among patients with DMD. We found that timed function tests other than 4SC, measures of walking and rising ability in particular, along with height, weight, and BMI added significant prognostic value for change in 4SC—well beyond that provided by age, baseline 4SC, and steroid duration. The proportion of variation explained by the addition of these factors was approximately doubled across all data sources.

Recently, we assessed prognostic factors for 1-year change in 6MWD in an earlier study sample of patients from the Leuven database [12]. A key finding of that study was that a combination of multiple baseline measures of ambulatory function provided significantly enhanced prognostic accuracy compared with using only age, baseline 6MWD, and steroid use. These findings are echoed in the present study of 4SC outcomes, further highlighting the value of combining multiple measures of function into composite prognostic scores in DMD. In addition, consistent with the prior study of prognostic factors for 6MWD, combining multiple measures of baseline function increases prognostic value even as those baseline measures are well correlated with each other [12]. Both of these studies also found that patient age no longer carries significant prognostic value after already accounting for multiple measures of baseline function along with height and weight. This indicates that knowing how a patient performs on several different measures of ambulatory function is more important to their prognosis over the next year than knowing their age. While this may at first seem counter-intuitive for a progressive disease, the lower prognostic importance of age accords with the observed heterogeneity across patients in rates of progression of ambulatory function in DMD. Different patients progress at different ages [20], and different measures of function progress at different times, and thus combining several measures of ambulatory function provides a more accurate prognosis than relying on any single measure alone, or on age.

Inclusion criteria for clinical trials in ambulatory DMD have traditionally been defined based on three conventional prognostic factors: age, steroid use for at least a defined period of time (3 or 6 months), and the baseline value of the primary endpoint (e.g., 6MWD or 4SC) [5–8, 21]. As the natural history of DMD has become better characterized, the ranges of these characteristics have varied, and generally narrowed [5, 22], with the aim of defining a subset of patients with more homogenous rates of progression in whom power to demonstrate treatment effects would be increased. In addition, primary analyses of clinical trials have typically adjusted for these conventional baseline factors (age, steroid use, and baseline assessments of the primary endpoint) in an effort to improve precision of estimated treatment effects. The present study is the second to demonstrate that composite prognostic scores can more than double the prognostic accuracy versus that provided by the conventional factors [12]. This indicates that composite prognostic scores for DMD present important opportunities for improving trial design and analyses. Many decisions in trial design are ultimately aimed at managing variation in outcomes or enriching for drug-modifiable patients. The more accurately one can predict outcomes, the better one can manage outcome variation and enrichment. Thus, these trial design decisions can be improved by use of an accurate prognostic score. For example, enrichment of a trial population (or a pre-specified subgroup) for patients with more homogenous and drug-modifiable trajectories will be more precise when the inclusion/exclusion criteria used to define the population are based on thresholds of an accurate composite prognostic score, instead of thresholds of individual characteristics with substantially lower prognostic value. When selecting baseline characteristics for stratifying randomization or for adjustment in statistical analyses of the treatment effect, it is usually not practical to stratify or adjust for more than 2 or 3 baseline characteristics. The stronger the prognostic factors that can be used, the greater the increase in power via stratification and adjustment. Use of a single composite prognostic score that already incorporates multiple baseline characteristics and doubles explained variation would be statistically superior to use of any combination of age, baseline 4SC, and steroid use.

Apart from parallel group randomized trials, a better understanding of prognostic factors for functional outcomes in DMD is needed to guide matched or adjusted comparisons to external control groups for single arm trials and long-term extension studies, and for augmentation of randomized placebo groups with external controls. A key step in the incorporation of external controls is mitigating the risk of bias due to differences in patient characteristics between non-randomized groups. It is usually not feasible to match on or adjust for *all* characteristics. Rather, the goal is to account for baseline differences in important prognostic factors. Thus, knowledge of important prognostic factors, or validated prognostic scores, is essential for making credible comparisons to external controls—and for avoiding the infeasibility or reductions in power that could arise from a desire to adjust for factors that are actually not prognostic.

This study has several strengths beginning with the inclusion of data from more than 350 boys diagnosed with DMD (n = 371) from three different sources. In addition, the general consistency of findings across data sources bodes well for the future construction of a well-validated prognostic score that performs well across data sources and can thus be reliably applied to clinical trials. In particular, despite known differences in specific measures between sites, such as differences in timed function test availability and assessment protocols, similar improvements in prognostic performance were observed when timed function tests were incorporated into the models. The use of different timed function tests in CCHMC in particular, i.e., 30 foot walk/run and sit to stand rather than 10 meter walk/run and rise from supine, qualitatively validates the models developed using the other data sources, and suggests that these tests are capturing aspects of patients' function that are biologically and statistically meaningful for prognosis.

In some cases, however, the differences across the data sources included in this study present some limitations. As discussed in the methods section we did not have explicit recording of loss of stair climbing ability in the CCHMC data used for these analyses, and both real-world data sources seemed to halt timed function tests earlier than observed in the clinical trial placebo arm. It should also be noted that the Tadalafil DMD Trial imposed stricter inclusion criteria of baseline 6MWD between 200 and 400 meters and >6 months stable steroid use [5], whereas the study samples for the other data sources required only 4SC time < 12 seconds. This is reflected by patients in the Tadalafil DMD Trial having worse performance on baseline functional measures, and a larger average decline in 4SC over the 1-year period, compared with the other sources. The real-world data sources also included more heterogeneous intervals of time between visits than the clinical trial. We conducted a number of sensitivity analyses to account for these differences across data sources, including use of different 4SC cutoffs (12 and 30 seconds), studying both 4SC completion time and velocity, and considering different windows of follow-up time to approximate 1-year change (8–16 months and 10–14 months). Our main findings were robust to these changes.

This study has demonstrated that composite prognostic scores for 4SC are likely to be feasible and to be important for clinical trial design, and that development of a consensus-based composite prognostic score is warranted. Additional prognostic factors should also be investigated, and might include other functional measures such as the NSAA [23–25] and measures of muscle strength, which have been correlated with functional status [26]. Imaging assessments of bone density and changes in lean body mass [27], cardiac and pulmonary measures [28, 29], dystrophin genotypes, genetic modifiers and other biomarkers could also be investigated in future analyses. Use of a prognostic score in clinical trial design and interpretation requires several steps beyond those conducted in the present study. Most importantly, a consensus score, ideally based on multiple data sources, will need to be developed and validated, and achieve clinical acceptance. In addition, the small (but not negligible) effort that it would take to calculate and interpret the score will need to be viewed as worthwhile relative to the gains that can be achieved in power and/or lower sample size requirements. Research to address these steps is currently being conducted within cTAP.

## Supporting information

S1 Table Intermediate models for Δ4SC velocity in each data source. (DOCX)

S2 Table a-c. R2 tables for Δ4SC velocity for (a) Tadalafil DMD Trial, (b) Leuven and (c) CCHMC.
(DOCX)

S3 Table Models for Δ4SC time in each data source. (DOCX)

S4 Table Intermediate models for Δ4SC time in each data source. (DOCX)

S5 Table a-c R^2^ tables for Δ4SC time for (a) Tadalafil DMD trial, (b) Leuven, and (c) CCHMC.
(DOCX)

S6 Table a. R^2^ in base, intermediate and full models for Δ4SC velocity based on primary and sensitivity analyses, by data source. b. R^2^ in base, intermediate and full models for Δ4SC time based on primary and sensitivity analyses, by data source.
(DOCX)

S1 Fig Scatter plots for observed versus predicted Δ4SC velocity for base and full model in each data source. (DOCX)

S2 Fig Observed Δ4SC velocity stratified by baseline prediction quartiles of Δ4SC velocity for base and full model in each data source. (DOCX)

S3 Fig Power to detect a treatment effect of 0.25 stairs/second with and without adjustment for prognostic score under different trial arm sample sizes, assuming the SD of Δ4SC velocity is 0.5. (DOCX)

S4 Fig Power to detect a treatment effect of 0.25 stairs/second with and without adjustment for prognostic score under different trial arm sample sizes, assuming the SD of Δ4SC velocity is 0.7. (DOCX)
