# Supplementary material for: Prognostic factors for changes in the timed 4-stair climb in patients with Duchenne muscular dystrophy, and implications for measuring drug efficacy: A multi-institutional collaboration
Source: PLoS One. 2020 Jun 18;15(6):e0232870. doi: 10.1371/journal.pone.0232870 (PMC7302444; doi:10.1371/journal.pone.0232870)
Supplement: S1 Table — (DOCX) [file pone.0232870.s001.docx]

## S1 Table. Intermediate models for ∆4SC velocity in each data source.

|  | Tadalafil DMD Trial Placebo Arm (n = 92) | Leuven (n = 235) | CCHMC (n = 543) |
| --- | --- | --- | --- |
|  | **Coefficient (95% CI)** | **Coefficient (95% CI)** | **Coefficient (95% CI)** |
| Intercept | -1.32 (-2.13, -0.50)** | 0.31 (0.03, 0.60)** | -0.13 (-0.44, 0.19) |
| Age (years) | 0.04 (-0.01, 0.08) | -0.05 (-0.07, -0.02)*** | -0.03 (-0.06, -0.01)* |
| Steroids ≥ 1 year 1 vs. 0 | 0.32 (0.09, 0.55)** | -0.29 (-0.46, -0.12)*** | -0.28 (-0.42, -0.15)*** |
| Timed 4SC (velocity) (stairs/second) | -0.17 (-0.68, 0.34) | -0.6 (-0.73, -0.47)*** | -0.48 (-0.56, -0.40)*** |
| Current deflazacort 1 vs. 0 | 0.18 (0.03, 0.33)* | 0.18 (0.01, 0.35) * | -0.01 (-0.14, 0.12) |
| Timed 10 meter walk/run (velocity) (meters/seconds) | 0.2 (-0.08, 0.49) | 0.28 (0.18, 0.38)*** | - |
| Timed rise from supine (velocity) (1/seconds) | 1.52 (0.52, 2.51)** | 1.87 (1.20, 2.55)*** | - |
| Timed 30 foot walk/run (velocity) (feet/seconds) | - | - | 0.15 (0.10, 0.20)*** |
| Timed sit to stand (velocity) (1/seconds) | - | - | 0.84 (0.44, 1.24)*** |
| Model R^2^ | 0.23 | 0.35 | 0.28 |
| RMSE | 0.36 | 0.52 | 0.48 |

∆4SC, annualized change in 4-stair climb; CCHMC, Cincinnati Children's Hospital Medical Center; CI, confidence interval; cm, centimeters; DMD, Duchenne muscular dystrophy; n, number of participants; RMSE, root-mean squared error.
∆4SC velocity = (4SC velocity at outcome visit - 4SC velocity at baseline visit)/ time in years between outcome and baseline visits. ∆4SC velocity > 0 indicates improved performance; ∆4SC velocity < 0 indicates worsened performance

Statistical significance: *** p < 0.001, ** p < 0.01, * p <0.05
