# Supplementary material for: Prognostic factors for changes in the timed 4-stair climb in patients with Duchenne muscular dystrophy, and implications for measuring drug efficacy: A multi-institutional collaboration
Source: PLoS One. 2020 Jun 18;15(6):e0232870. doi: 10.1371/journal.pone.0232870 (PMC7302444; doi:10.1371/journal.pone.0232870)
Supplement: S2 Table — a-c. R2 tables for Δ4SC velocity for (a) Tadalafil DMD Trial, (b) Leuven and (c) CCHMC. (DOCX) [file pone.0232870.s002.docx]

## S2a-2c Table. R^2^ tables for ∆4SC velocity for (a) Tadalafil DMD Trial, (b) Leuven, and (c) CCHMC.

|  | **Removed from full model** | **Added to base model** | **Studied in isolation** |
| --- | --- | --- | --- |
| **Tadalafil DMD Trial** | **(Reference = 0.29)** | **(Reference = 0.08)** | **(Reference = 0.0)** |
| Age (years) | 0.28 | 0.08 | 0 |
| Steroids ≥ 1 year | 0.26 | 0.08 | 0 |
| Timed 4SC (velocity) (stairs/second) | 0.28 | 0.08 | 0.07 |
| Current deflazacort | 0.25 | 0.14 | 0.06 |
| Timed 10MWR (velocity) (meters/seconds) | 0.27 | 0.12 | 0.11 |
| Timed rise from supine (velocity) (1/seconds) | 0.25 | 0.18 | 0.15 |
| BMI (kg/m^2^) | 0.24 | 0.09 | 0.01 |
| Height (cm) | 0.24 | 0.09 | 0.02 |
| Weight (kg) | 0.24 | 0.09 | 0.02 |

|  | **Removed from full model** | **Added to base model** | **Studied in isolation** |
| --- | --- | --- | --- |
| **Leuven** | **(Reference = 0.36)** | **(Reference = 0.17)** | **(Reference = 0.0)** |
| Age (years) | 0.36 | 0.17 | 0.11 |
| Steroids ≥ 1 year | 0.34 | 0.17 | 0.04 |
| Timed 4SC (velocity) (stairs/second) | 0.14 | 0.17 | 0.04 |
| Current deflazacort | 0.36 | 0.19 | 0.01 |
| Timed 10MWR (velocity) (meters/seconds) | 0.32 | 0.29 | 0.03 |
| Timed rise from supine (velocity) (1/seconds) | 0.31 | 0.3 | 0.03 |
| BMI (kg/m^2^) | 0.36 | 0.17 | 0.03 |
| Height (cm) | 0.36 | 0.2 | 0.11 |
| Weight (kg) | 0.36 | 0.17 | 0.06 |

|  | **Removed from full model** | **Added to base model** | **Studied in isolation** |
| --- | --- | --- | --- |
| **CCHMC** | **(Reference = 0.30)** | **(Reference = 0.16)** | **(Reference = 0.0)** |
| Age (years) | 0.29 | 0.16 | 0.1 |
| Steroids ≥ 1 year | 0.29 | 0.16 | 0.03 |
| Timed 4SC (velocity) (stairs/second) | 0.17 | 0.16 | 0.05 |
| Current deflazacort | 0.3 | 0.16 | 0.01 |
| Timed 30 foot walk/run (velocity) (feet/seconds) | 0.26 | 0.25 | 0 |
| Timed sit to stand (velocity) (1/seconds) | 0.29 | 0.23 | 0.02 |
| BMI (kg/m^2^) | 0.28 | 0.16 | 0.03 |
| Height (cm) | 0.29 | 0.16 | 0.08 |
| Weight (kg) | 0.28 | 0.16 | 0.05 |

∆4SC, annualized change in 4-stair climb; 10MWR, 10-meter walk/run; BMI, body mass index; CCHMC, Cincinnati Children's Hospital Medical Center; cm, centimeters; DMD, Duchenne muscular dystrophy; kg, kilogram; m^2^, meter squared.
