# Supplementary material for: Prognostic factors for changes in the timed 4-stair climb in patients with Duchenne muscular dystrophy, and implications for measuring drug efficacy: A multi-institutional collaboration
Source: PLoS One. 2020 Jun 18;15(6):e0232870. doi: 10.1371/journal.pone.0232870 (PMC7302444; doi:10.1371/journal.pone.0232870)
Supplement: S3 Table — (DOCX) [file pone.0232870.s003.docx]

## S3 Table. Models for ∆4SC time in each data source.

|  | Tadalafil DMD Trial Placebo Arm (n = 92) | | Leuven (n = 235) | | CCHMC (n = 543) | |
| --- | --- | --- | --- | --- | --- | --- |
|  | Base model | Full model | Base model | Full model | Base model | Full model |
|  | Coefficient (95% CI) | Coefficient (95% CI) | Coefficient (95% CI) | Coefficient (95% CI) | Coefficient (95% CI) | Coefficient (95% CI) |
| Intercept | 2.49 (-3.32, 8.30) | -40.62 (-76.67, -4.57)* | -2.15 (-3.01, -1.29)*** | -5.28 (-16.22, 5.65) | -1.49 (-2.10, -0.89)*** | -14.41 (-22.47, -6.36)*** |
| Age (years) | -0.15 (-0.62, 0.32) | -0.26 (-0.69, 0.17) | 0.21 (0.10, 0.32) *** | -0.04 (-0.20, 0.12) | 0.15 (0.09, 0.21)*** | 0.09 (0.00, 0.18)* |
| Steroids ≥ 1 year 1 vs. 0 | 0 (-2.33, 2.33) | -1.71 (-3.69, 0.27) | 0.82 (0.31, 1.33) ** | 1.26 (0.66, 1.86) *** | 0.44 (0.11, 0.77)** | 0.69 (0.32, 1.06)*** |
| Timed 4SC (seconds) | 0.18 (-0.18, 0.55) | -0.3 (-0.73, 0.13) | 0.07 (-0.01, 0.16) | -0.44 (-0.63, -0.24) *** | 0.1 (-0.05, 0.25) | -0.52 (-0.73, -0.31) *** |
| Current deflazacort 1 vs. 0 |  | -1.51 (-2.65, -0.37)** |  | -0.29 (-0.91, 0.33) |  | -0.19 (-0.54, 0.17) |
| Timed 10-meter walk/run (seconds) |  | 0.54 (-0.10, 1.17) |  | 0.28 (0.05, 0.51) * | - | - |
| Timed rise from supine (seconds) |  | 0.17 (-0.04, 0.39) |  | 0.3 (0.16, 0.44) *** | - | - |
| Timed 30-foot walk/run (seconds) | - | - | - | - |  | 0.64 (0.36, 0.92)*** |
| Timed sit to stand (seconds) | - | - | - | - |  | 0.3 (0.15, 0.45)*** |
| BMI (kg/m^2^) |  | 1.21 (0.29, 2.14)* |  | -0.09 (-0.38, 0.20) |  | 0.3 (0.11, 0.49)** |
| Height (cm) |  | 0.34 (0.05, 0.62)* |  | 0.04 (-0.05, 0.13) |  | 0.09 (0.03, 0.16)** |
| Weight (kg) |  | -0.7 (-1.27, -0.12)* |  | 0.03 (-0.13, 0.18) |  | -0.2 (-0.33, -0.07)** |
| Model R^2^ | 0.02 | 0.29 | 0.13 | 0.34 | 0.11 | 0.34 |
| RMSE | 2.81 | 2.39 | 2.05 | 1.79 | 1.43 | 1.23 |

∆4SC, annualized change in 4-stair climb; BMI, body mass index; CCHMC, Cincinnati Children's Hospital Medical Center; CI, confidence interval; cm, centimeters; DMD, Duchenne muscular dystrophy; kg, kilograms; m^2^, meters squared; n, number of intervals; RMSE, root-mean squared error.

∆4SC time = (4SC time at outcome visit - 4SC time at baseline visit)/ time in years between outcome and baseline visits. ∆4SC time > 0 indicates worsened performance; ∆4SC time < 0 improved performance.
Statistical significance: *** p < 0.001, ** p < 0.01, * p <0.05.
