# Supplementary material for: Prognostic factors for changes in the timed 4-stair climb in patients with Duchenne muscular dystrophy, and implications for measuring drug efficacy: A multi-institutional collaboration
Source: PLoS One. 2020 Jun 18;15(6):e0232870. doi: 10.1371/journal.pone.0232870 (PMC7302444; doi:10.1371/journal.pone.0232870)
Supplement: S4 Table — (DOCX) [file pone.0232870.s004.docx]

## S4 Table. Intermediate models for ∆4SC time in each data source.

|  | Tadalafil DMD Trial Placebo Arm (n = 92) | Leuven (n = 235) | CCHMC (n = 543) |
| --- | --- | --- | --- |
|  | **Coefficient (95% CI)** | **Coefficient (95% CI)** | **Coefficient (95% CI)** |
| Intercept | 2.31 (-2.97, 7.60) | -2.52 (-3.42, -1.63)*** | -2.79 (-3.94, -1.63)*** |
| Age (years) | -0.21 (-0.62, 0.19) | 0.09 (-0.01, 0.18) | 0.05 (-0.01, 0.12) |
| Steroids ≥ 1 year 1 vs. 0 | -1.19 (-3.07, 0.69) | 1.22 (0.62, 1.83)*** | 0.78 (0.40, 1.15)*** |
| Timed 4SC (seconds) | -0.31 (-0.77, 0.14) | -0.45 (-0.65, -0.26)*** | -0.57 (-0.79, -0.35)*** |
| Current deflazacort 1 vs. 0 | -1.63 (-2.72, -0.53)** | -0.49 (-1.14, 0.15) | -0.16 (-0.54, 0.22) |
| Timed 10 meter walk/run (seconds) | 0.55 (-0.11, 1.21) | 0.33 (0.09, 0.57)** | - |
| Timed rise from supine (seconds) | 0.21 (-0.01, 0.43) | 0.31 (0.16, 0.46)*** | - |
| Timed 30 foot walk/run (seconds) | - | - | 0.65 (0.35, 0.94)*** |
| Timed sit to stand (seconds) | - | - | 0.33 (0.17, 0.49)*** |
| Model R^2^ | 0.24 | 0.32 | 0.29 |
| RMSE | 2.48 | 1.81 | 1.28 |

∆4SC, annualized change in 4-stair climb; CCHMC, Cincinnati Children's Hospital Medical Center; CI, confidence interval; DMD, Duchenne muscular dystrophy; n, number of participants; RMSE, root-mean squared error.

∆4SC time = (4SC time at outcome visit - 4SC time at baseline visit)/ time in years between outcome and baseline visits. ∆4SC time > 0 indicates worsened performance; ∆4SC time < 0 improved performance.
Statistical significance: *** p < 0.001, ** p < 0.01, * p <0.05.
