# Supplementary material for: Prognostic factors for changes in the timed 4-stair climb in patients with Duchenne muscular dystrophy, and implications for measuring drug efficacy: A multi-institutional collaboration
Source: PLoS One. 2020 Jun 18;15(6):e0232870. doi: 10.1371/journal.pone.0232870 (PMC7302444; doi:10.1371/journal.pone.0232870)
Supplement: S5 Table — a-c R2 tables for Δ4SC time for (a) Tadalafil DMD trial, (b) Leuven, and (c) CCHMC. (DOCX) [file pone.0232870.s005.docx]

## S5a-5c Table. R^2^ tables for ∆4SC time for (a) Tadalafil DMD trial, (b) Leuven, and (c) CCHMC.

|  | **Removed from full model** | **Added to base model** | **Studied in isolation** |
| --- | --- | --- | --- |
| **Tadalafil DMD Trial** | **(Reference = 0.29)** | **(Reference = 0.02)** | **(Reference = 0.0)** |
| Age (years) | 0.28 | 0.02 | 0 |
| Steroids ≥ 1 year | 0.28 | 0.02 | 0 |
| Timed 4SC (seconds) | 0.27 | 0.02 | 0.01 |
| Current deflazacort | 0.23 | 0.12 | 0.11 |
| Timed 10MWR (seconds) | 0.25 | 0.13 | 0.09 |
| Timed rise from supine (seconds) | 0.27 | 0.11 | 0.08 |
| BMI (kg/m^2^) | 0.24 | 0.05 | 0.02 |
| Height (cm) | 0.25 | 0.02 | 0 |
| Weight (kg) | 0.25 | 0.05 | 0.02 |

| **Leuven** | **Removed from full model** | **Added to base model** | **Studied in isolation** |
| --- | --- | --- | --- |
|  | **(Reference = 0.34)** | **(Reference = 0.13)** | **(Reference = 0.0)** |
| Age (years) | 0.34 | 0.13 | 0.11 |
| Steroids ≥ 1 year | 0.32 | 0.13 | 0.06 |
| Timed 4SC (seconds) | 0.24 | 0.13 | 0.01 |
| Current deflazacort | 0.34 | 0.14 | 0.01 |
| Timed 10MWR (seconds) | 0.31 | 0.24 | 0.11 |
| Timed rise from supine (seconds) | 0.28 | 0.28 | 0.16 |
| BMI (kg/m^2^) | 0.34 | 0.13 | 0.03 |
| Height (cm) | 0.34 | 0.18 | 0.16 |
| Weight (kg) | 0.34 | 0.14 | 0.09 |

|  | **Removed from full model** | **Added to base model** | **Studied in isolation** |
| --- | --- | --- | --- |
| **CCHMC** | **(Reference = 0.34)** | **(Reference = 0.11)** | **(Reference = 0.0)** |
| Age (years) | 0.33 | 0.11 | 0.09 |
| Steroids ≥ 1 year | 0.32 | 0.11 | 0.02 |
| Timed 4SC (seconds) | 0.27 | 0.11 | 0.01 |
| Current deflazacort | 0.33 | 0.12 | 0 |
| Timed 30 foot walk/run (seconds) | 0.29 | 0.22 | 0.08 |
| Timed sit to stand (seconds) | 0.29 | 0.24 | 0.17 |
| BMI (kg/m^2^) | 0.3 | 0.11 | 0.02 |
| Height (cm) | 0.3 | 0.11 | 0.07 |
| Weight (kg) | 0.29 | 0.11 | 0.03 |

∆4SC, annualized change in 4-stair climb; 10MWR, 10-meter walk/run; BMI, body mass index; CCHMC, Cincinnati Children's Hospital Medical Center; cm, centimeters; DMD, Duchenne muscular dystrophy; kg, kilogram; m^2^, meters squared.
