# Supplementary material for: Prognostic factors for changes in the timed 4-stair climb in patients with Duchenne muscular dystrophy, and implications for measuring drug efficacy: A multi-institutional collaboration
Source: PLoS One. 2020 Jun 18;15(6):e0232870. doi: 10.1371/journal.pone.0232870 (PMC7302444; doi:10.1371/journal.pone.0232870)
Supplement: S6 Table — a. R2 in base, intermediate and full models for Δ4SC velocity based on primary and sensitivity analyses, by data source. b. R2 in base, intermediate and full models for Δ4SC time based on primary and sensitivity analyses, by data source. (DOCX) [file pone.0232870.s006.docx]

## S6a Table. R^2^ in base, intermediate and full models for ∆4SC velocity based on primary and sensitivity analyses, by data source.

|  |  | **Tadalafil DMD Trial** | **Leuven** | **CCHMC** |
| --- | --- | --- | --- | --- |
| Base model | Primary analysis | 0.08 | 0.17 | 0.16 |
|  | Sensitivity analysis 1 | 0.05 | 0.17 | 0.16 |
|  | Sensitivity analysis 2 | 0.08 | 0.18 | 0.15 |
| Intermediate model | Primary analysis | 0.23 | 0.35 | 0.28 |
|  | Sensitivity analysis 1 | 0.21 | 0.35 | 0.28 |
|  | Sensitivity analysis 2 | 0.23 | 0.34 | 0.27 |
| Full model | Primary analysis | 0.29 | 0.36 | 0.30 |
|  | Sensitivity analysis 1 | 0.25 | 0.36 | 0.31 |
|  | Sensitivity analysis 2 | 0.28 | 0.35 | 0.30 |

∆4SC, annualized change in 4-stair climb; CCHMC, Cincinnati Children's Hospital Medical Center; DMD, Duchenne muscular dystrophy.

Sensitivity analysis 1: 4SC times for patients who lost the ability to climb stairs were set to 30 seconds; patients with 4SC completion times exceeding 30 seconds had these times truncated to 30 seconds.
Sensitivity analysis 2: In this analysis, ~1-year follow-up intervals were defined as visits between 10 and 14 months apart.

## S6b Table. R^2^ in base, intermediate and full models for ∆4SC time based on primary and sensitivity analyses, by data source.

|  |  | **Tadalafil DMD Trial** | **Leuven** | **CCHMC** |
| --- | --- | --- | --- | --- |
| Base model | Primary analysis | 0.02 | 0.13 | 0.11 |
|  | Sensitivity analysis 1 | 0.01 | 0.12 | 0.10 |
|  | Sensitivity analysis 2 | 0.02 | 0.14 | 0.12 |
| Intermediate model | Primary analysis | 0.24 | 0.32 | 0.29 |
|  | Sensitivity analysis 1 | 0.24 | 0.31 | 0.31 |
|  | Sensitivity analysis 2 | 0.23 | 0.29 | 0.30 |
| Full model | Primary analysis | 0.29 | 0.34 | 0.34 |
|  | Sensitivity analysis 1 | 0.29 | 0.33 | 0.36 |
|  | Sensitivity analysis 2 | 0.28 | 0.31 | 0.35 |

∆4SC, annualized change in 4-stair climb; CCHMC, Cincinnati Children's Hospital Medical Center; DMD, Duchenne muscular dystrophy.

Sensitivity analysis 1: 4SC times for patients who lost the ability to climb stairs were set to 30 seconds; patients with 4SC completion times exceeding 30 seconds had these times truncated to 30 seconds.
Sensitivity analysis 2: In this analysis, ~1-year follow-up intervals were defined as visits between 10 and 14 months apart.
