# Supplementary material for: Prognostic factors for changes in the timed 4-stair climb in patients with Duchenne muscular dystrophy, and implications for measuring drug efficacy: A multi-institutional collaboration
Source: PLoS One. 2020 Jun 18;15(6):e0232870. doi: 10.1371/journal.pone.0232870 (PMC7302444; doi:10.1371/journal.pone.0232870)
Supplement: S1 Fig — (DOCX) [file pone.0232870.s007.docx]

## S1 Fig. Scatter plots for observed versus predicted ∆4SC velocity for base and full model in each data source.

| **Base model in Tadalafil DMD Trial**  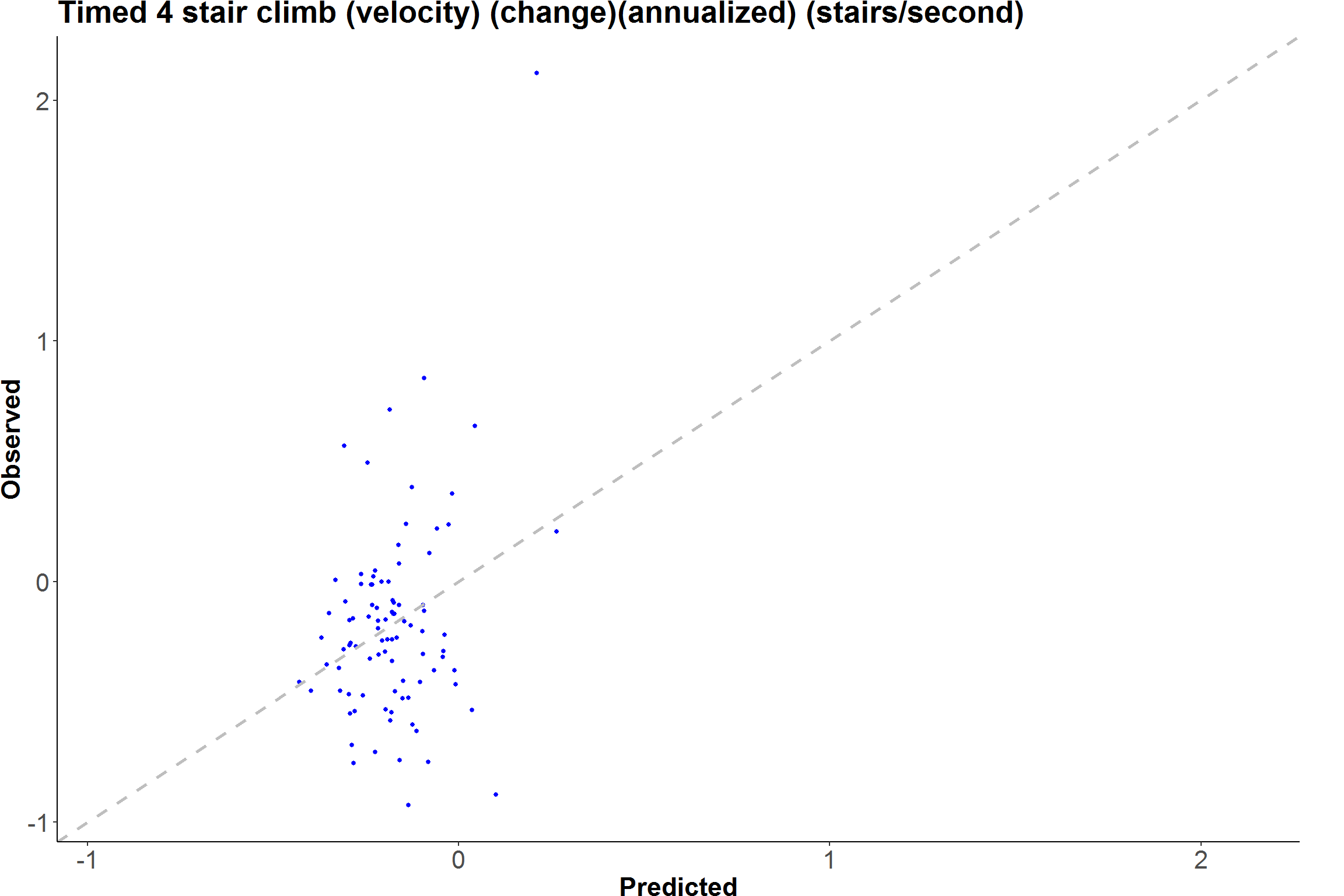 | **Full model in Tadalafil DMD Trial**  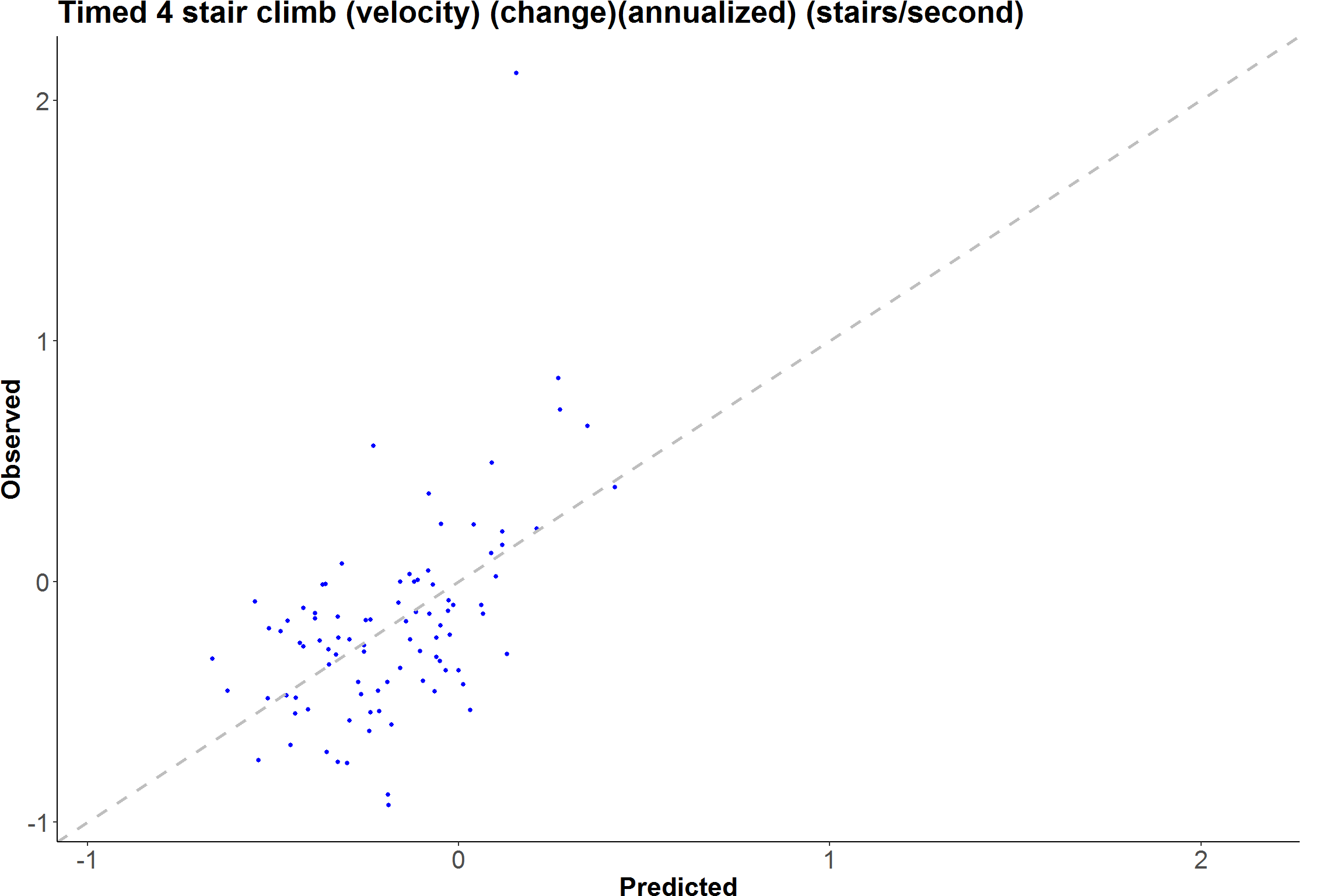 |
| --- | --- |
| **Base model in Leuven**  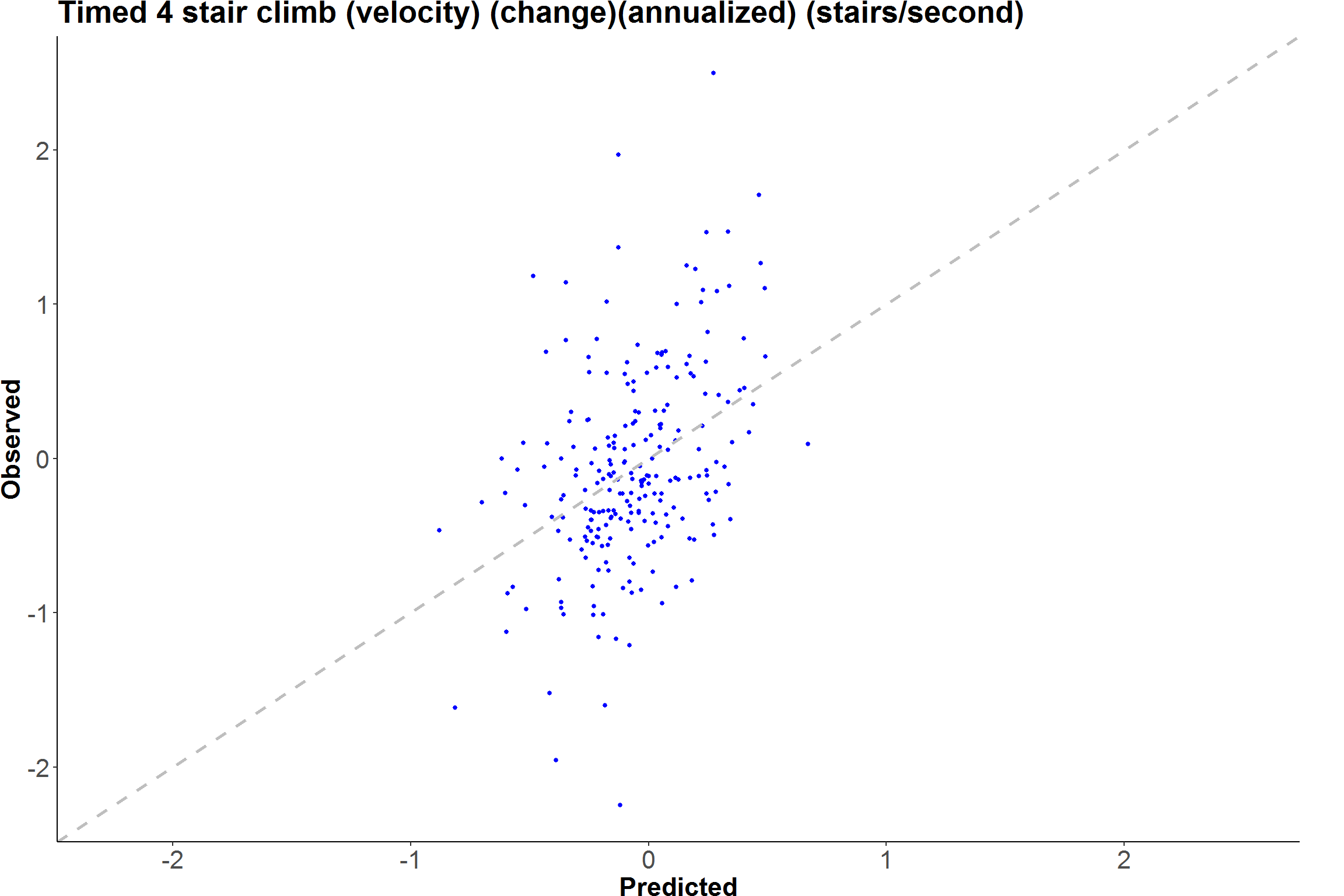 | **Full model in Leuven**  **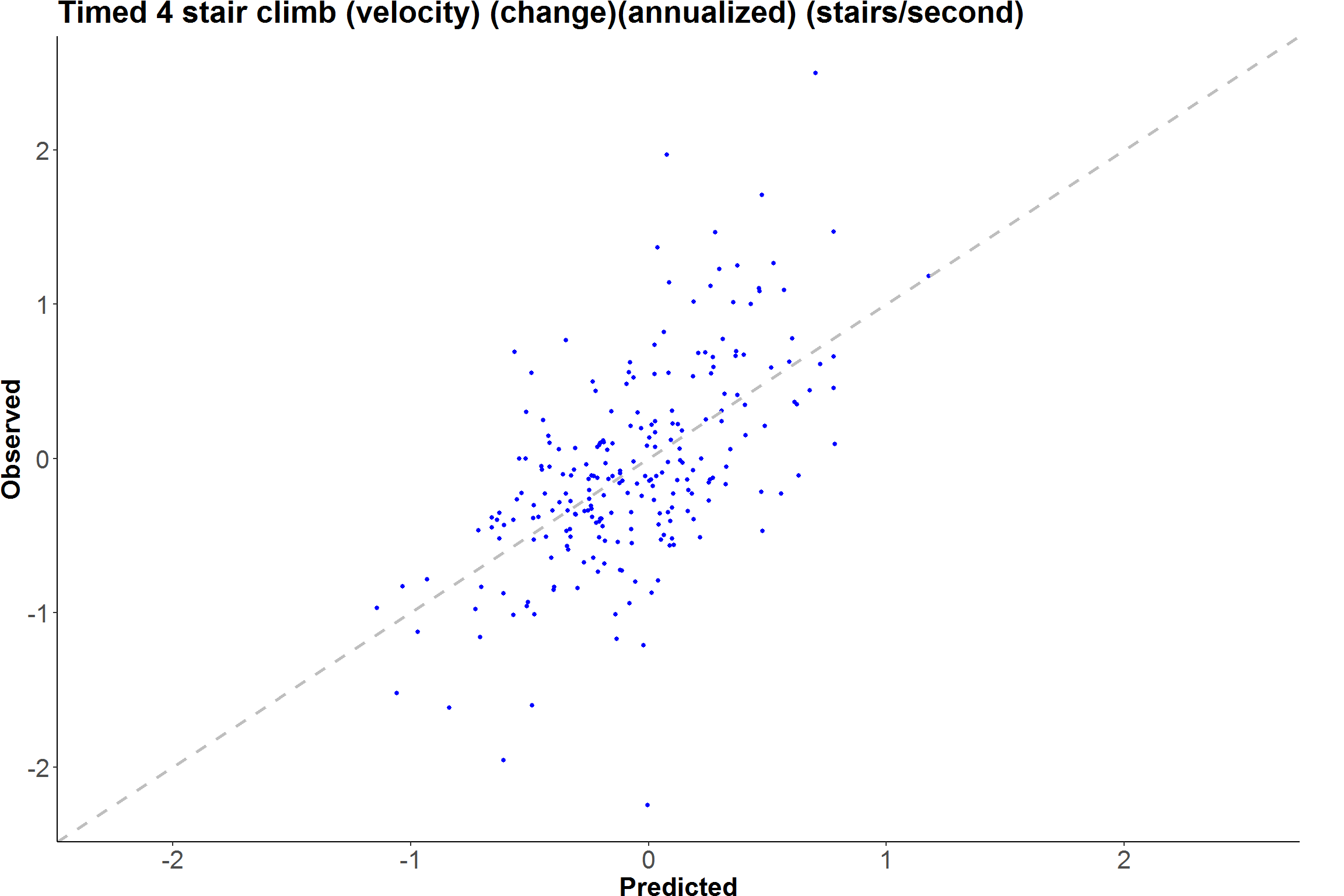** |
| **Base model in CCHMC**  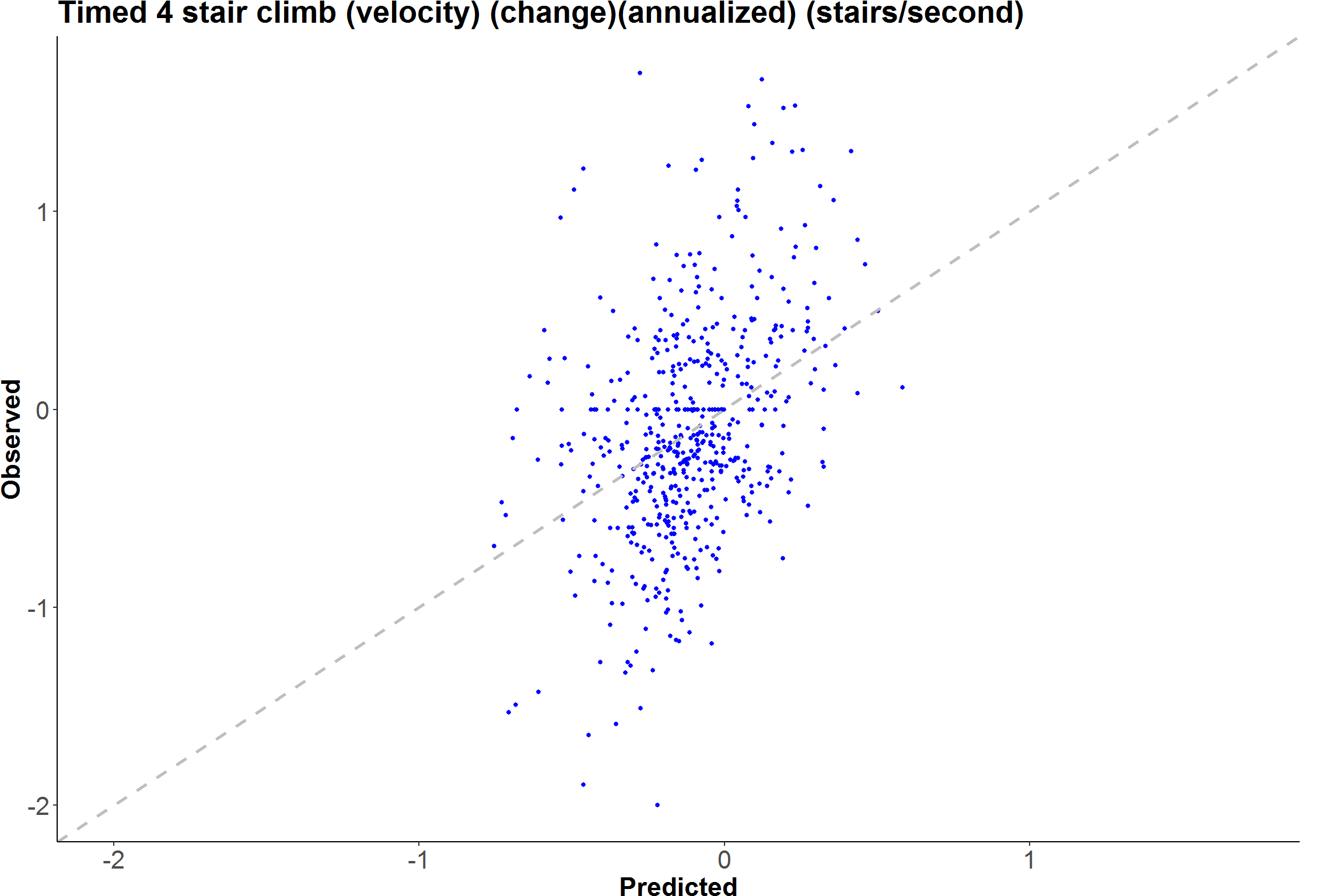 | **Full model in CCHMC**  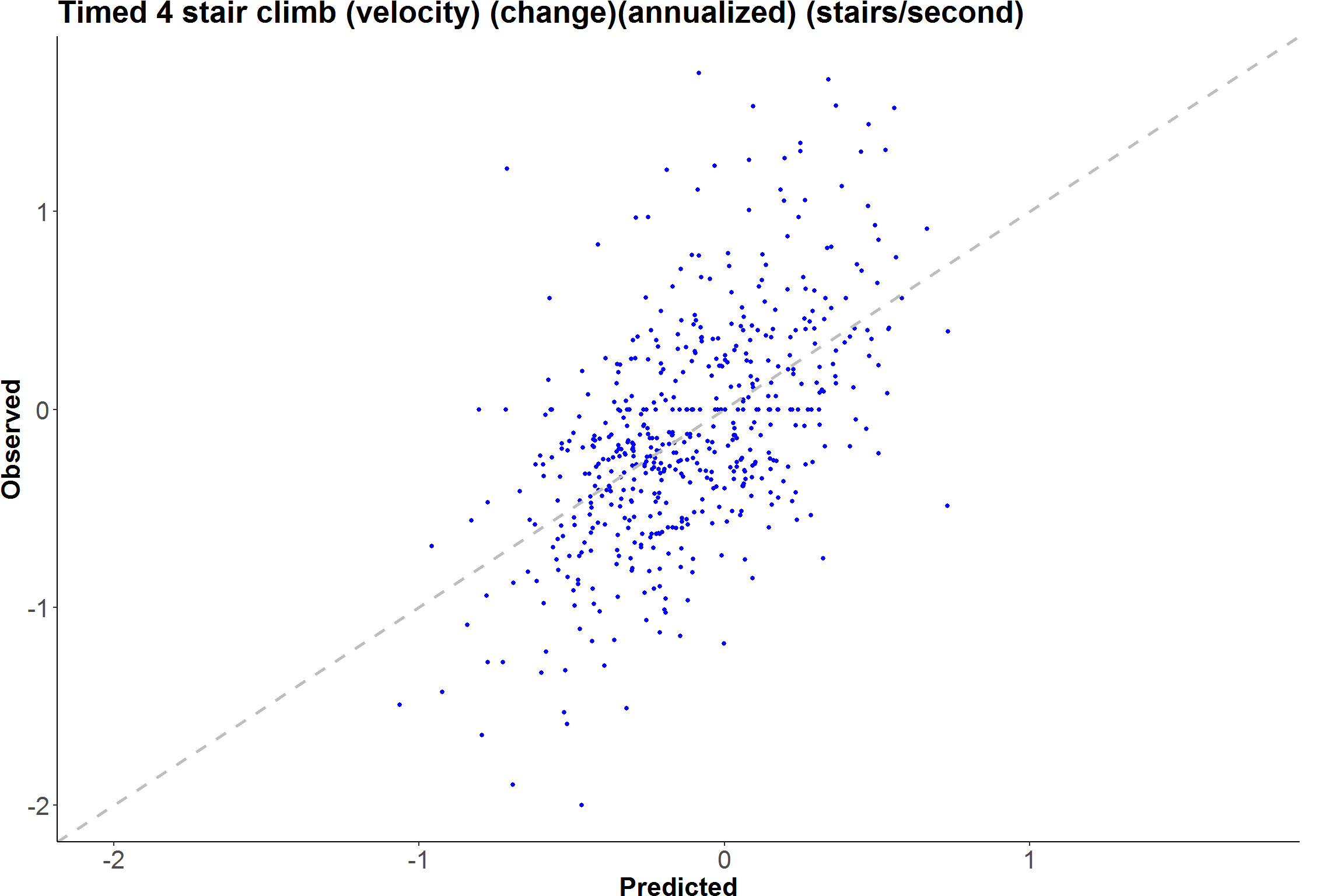 |

∆4SC, annualized change in 4-stair climb; CCHMC, Cincinnati Children's Hospital Medical Center; DMD, Duchenne muscular dystrophy.
