# Supplementary material for: Prognostic factors for changes in the timed 4-stair climb in patients with Duchenne muscular dystrophy, and implications for measuring drug efficacy: A multi-institutional collaboration
Source: PLoS One. 2020 Jun 18;15(6):e0232870. doi: 10.1371/journal.pone.0232870 (PMC7302444; doi:10.1371/journal.pone.0232870)
Supplement: S4 Fig — (DOCX) [file pone.0232870.s010.docx]

## S4 Fig. Power to detect a treatment effect of 0.25 stairs/second with and without adjustment for prognostic score under different trial arm sample sizes, assuming the SD of ∆4SC velocity is 0.7.


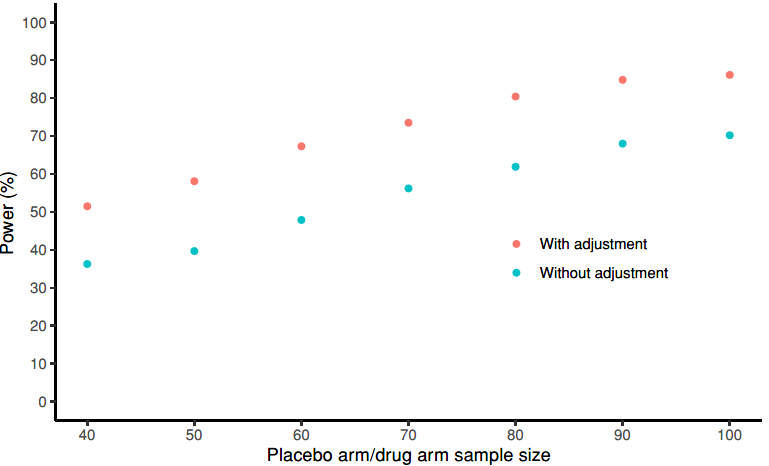


**∆**4SC, annualized change in 4-stair climb; SD, standard deviation. ∆4SC velocity = (4SC velocity at outcome visit - 4SC velocity at baseline visit)/ time in years between outcome and baseline visits. ∆4SC velocity > 0 indicates improved performance; ∆4SC velocity < 0 indicates worsened performance

Note: R-squared due to prognostic model assumed to be 0.35 in all scenarios. While a SD of ∆4SC velocity of 0.7 was used for this illustration, a similar pattern of greater power to detect a treatment effect when adjustment is made for the prognostic score also holds for other values of SDs of ∆4SC velocity (Fig 2 and S3 Fig).
